# Internet Development, Consumption Upgrading and Carbon Emissions—An Empirical Study from China

**DOI:** 10.3390/ijerph20010265

**Published:** 2022-12-24

**Authors:** Yingzi Chen, Wanwan Yang, Yaqi Hu

**Affiliations:** 1 Center for Northeast Asian Studies, Jilin University, Changchun 130012, China; 2 Northeast Asian College, Jilin University, Changchun 130012, China

**Keywords:** Internet development, consumption upgrading, carbon emissions, fixed effects model, mediating effects model, low-carbon consumption

## Abstract

Internet development has changed Chinese people’s consumption behavior, gradually expanding from survival consumption (SC) to development and enjoyment consumption (DEC) trends. Consumption is the new engine driving China’s economic growth and the terminal of carbon emissions. Simultaneously, China is undergoing a profound change toward the “double carbon” goal, the space for carbon emission reduction in traditional fields is gradually compressed. Therefore, it is necessary to explore carbon emissions from the perspective of consumption terminals. Based on provincial panel data, we use the fixed effects model and mediating effects model to explore the relationship between Internet development, consumption upgrading, and carbon emissions in a unified research framework. The findings show that: (1) Internet development leads to an increase in carbon emissions. A finding remains significant after using instrumental variables to mitigate endogeneity; (2) Internet development promotes consumption upgrading, reflected in development and enjoyment consumption expenditure; (3) Internet development contributes to increasing carbon emissions through consumption upgrading. Heterogeneity analysis shows that Internet development in eastern China significantly contributes to carbon emissions through consumption upgrading, while it is insignificant in central and western regions. The Internet development leading region contributes to an increase in carbon emissions through consumption upgrading. In comparison, the lagging region is insignificant. This study can provide a reference for policymakers in China or other countries to formulate energy-saving and emission-reduction policies in the Internet industry and provide a scientific basis for advocating people’s low-carbon consumption behavior and achieving carbon emission reduction at the consumption terminal.

## 1. Introduction

The development of the Internet has given vitality to thousands of industries and stimulated rapid economic growth in various fields worldwide. In 2021, the size of the digital economy in 47 countries worldwide was USD 38.1 trillion in value-added and 45.0% of GDP [1]. As a result, the Internet economy is becoming the new engine of global economic development. Reducing carbon emissions and promoting green development have become common global concerns as the global climate is getting warmer. Therefore, in the context of the “carbon neutrality goal”, it is necessary to study the relationship between Internet development and carbon emissions. For example, the carbon-intensive inputs of the Internet industry through other sectors may lead to changes in carbon emissions [2]. As the space for carbon reduction in traditional carbon-intensive sectors is gradually being compressed, it becomes significant to explore carbon emissions in the consumption sector.

Internet development has extensively changed China’s consumption pattern and consumption environment and Chinese people’s consumption behavior is transitioning from SC to a new consumption structure stage of DEC, i.e., consumption upgrading. China leads the world in terms of its Internet user base and consumer market size. According to the 47th China Internet Development Statistics Report, as of December 2020, the size of China’s Internet users was 989 million, the Internet penetration rate reached 70.4%, and the size of online shopping users reached 782 million. This shows that the Chinese consumer market has become highly digitalized. However, the carbon emissions of data centers, which are responsible for the operation of Internet technology, are increasing, and China’s data center carbon emissions as a percentage of total carbon emissions are twice as high as the global average [3]. Therefore, China’s 14th Five-Year Plan for developing the digital economy calls for “continuously promoting the construction of green digital centers following the principles of green, low-carbon, intensive and efficient” [4]. An effective combination of Internet development and carbon emissions is urgently needed to achieve the global goal of carbon neutrality. Therefore, there is a need to understand whether Internet development affects carbon emissions. If Internet development affects carbon emissions, does consumption become a transmission mechanism? This is a question that deserves to be explored in depth. The existing literature has mainly explored the impact of Internet development on carbon emissions. However, few have further elaborated on the transmission mechanism between the two [5], especially the need to explore the relationship between Internet development and carbon emissions from the perspective of consumption upgrading. To this end, we selected a study sample of 30 Chinese provinces (except Tibet, Hong Kong, Macao, and Taiwan) and used data from 2000 to 2018. We analyzed the direct and indirect effects of Internet development on carbon emissions and further explored consumption upgrading as a transmission mechanism between Internet development and carbon emissions using a mediating effects model. The article’s ideas may be helpful for low-carbon development and low-carbon consumption in China and other countries.

The possible innovations of this study are as follows. First, differing from the existing literature, this paper explores the impact of Internet development on carbon emissions by considering the breadth and depth of the Internet, which avoids the problem of a single selection of indicators. Second, the study’s findings provide new explanations for the impact of the Internet on carbon emissions. Contrary to the conclusion that Internet development promotes low-carbon development, we find that Internet development increases carbon emissions. This finding enriches the relationship between the impact of carbon emissions. More importantly, it can make policymakers pay attention to the negative impact of Internet development on carbon emissions. Third, this study integrates the application of sociological and psychological-related theories to explore the transmission mechanism between Internet development and carbon emissions when consumption upgrading is a mediating variable. The case of China validates this finding and this finding complements theoretical and empirical studies focusing on traditional transmission mechanisms.

**Figure 1:**
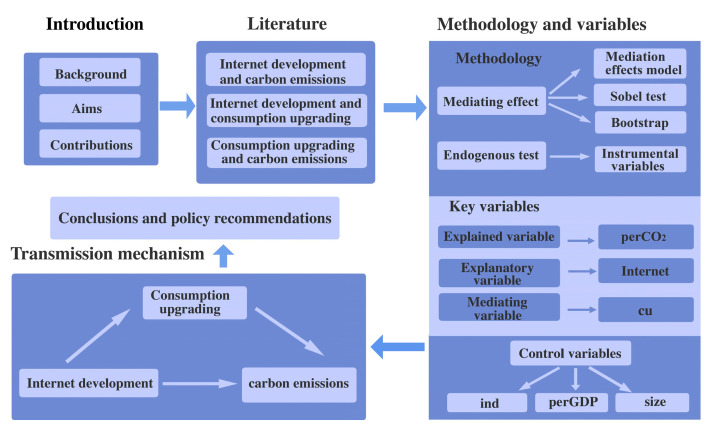
Analysis framework.

The remainder of the paper is structured as follows. Section 2 provides a literature review, theoretical analysis, and research hypotheses. Section 3 briefly explains the methodology and data used in this research. Section 4 describes the empirical results. Section 5 presents the discussion section. Section 6 provides conclusions and policy recommendations. Figure 1 shows the analysis framework of the article.

## 2. Literature Review

### 2.1. Internet Development and Carbon Emissions

The rapid growth of the Internet has dramatically changed the global economy, society, and the environment. Internet technology applications penetrate and rapidly connect other economic sectors. Internet development can increase the efficiency of production and consumption processes and is an essential driver of economic growth [6]. Numerous studies have shown that Internet development, communication technologies, and effective government governance are critical indicators for reducing carbon emissions [7,8,9,10,11]. Internet development mainly improves energy and carbon emission performance by promoting industrial structure upgrading and technology diffusion [12]. The deep integration of Internet technologies with various industries is beneficial for carbon emission reduction, such as in the green and low-carbon transformation of manufacturing [13], transportation [14], and agriculture [15].

Internet activities have been closely related to human activities during the past decade and profoundly impacted individuals’ environmental protection behavior. Some studies have shown that Internet use can directly influence individual consciousness, consumption behavior, and lifestyle [16,17] and promote the emergence of individual pro-environment behaviors. For example, studies show that Internet development impacts consumer behavior, reducing household electricity consumption by about 0–5% [18]. In addition, the widespread use of Internet technology has changed traditional business and consumption patterns. The rapid development of e-commerce and online platforms has reduced the investment in the decoration of physical stores, reducing the demand for coal and cement and reducing carbon emissions.

However, while Internet development reduces carbon emissions, as described above, it can also contribute to increased carbon emissions. Internet technologies themselves rely on energy-intensive infrastructure to operate. The heavy use of equipment associated with Internet technologies (e.g., computers and data centers) will lead to more energy consumption, especially the high demand for electricity, which is one of the critical sources of global carbon emissions [19,20]. If the Internet industry grows rapidly without limit, its carbon emissions will exceed 16% of the global carbon emissions in 2016 by 2040, with data center emissions accounting for 45% [21]. Currently, a large proportion of European domestic electricity consumption is related to Internet-related products and services [22]. Future Internet development in China may contribute to about 6% of China’s carbon emissions [23]. Scholars studied the impact of Internet technologies on CO_2_ emissions in China, Southeast Asian countries, and emerging countries [5,24,25], combining Internet technologies with economics and finance, and have similarly concluded that carbon emissions increase. Additionally, some scholars have suggested an inverted U-shaped relationship between Internet technology and carbon emissions, indicating that the turning point of Internet development is higher in developing countries than in developed countries [26]. A significant limitation of these studies is that they focus on carbon emissions generated by the operation of Internet technologies themselves. A few involve Internet-related economic activities but still explore the impact of Internet development on carbon emissions from traditional transmission perspectives, such as industrial structure [27,28], technology diffusion [12], energy structure [29], and energy efficiency [24]. However, with the development of the Internet, people’s consumption patterns have changed. The impact of consumption growth and consumption upgrading on carbon emissions cannot be ignored. The impact of this Internet-driven consumption upgrading on carbon emissions has yet to be considered in scholars’ studies.

### 2.2. The Impact of Internet Development on Consumption Upgrading

“Consumption upgrading” refers to the proportion of DEC expenditure to total consumption expenditure and generally refers to consumption structure upgrading. Consumption upgrading directly reflects the trend of consumption tendency and economic development trend, which is one of the essential features of the Internet era and an important element of terminal carbon emission structure adjustment.

Internet development has profoundly affected people’s daily consumption patterns. Internet use has influenced consumers’ attitudes and behaviors toward purchasing goods [30,31]. Internet use has significantly increased household consumption expenditures [32] and Internet access has facilitated residential consumption upgrading [33,34]. At the same time, Internet development has changed financial service patterns and payment methods. Some studies have shown that the impact of digital finance on household consumption structure cannot be ignored [35,36,37,38,39,40,41] and mobile payment is one-factor influencing household consumption [42,43,44]. The development of the Internet economy affects the consumption of different age groups, such as adolescents [31] and the elderly [45]. In addition, some scholars have explored the impact of the Internet on green consumption views and attitudes. Füller et al. (2009) argued that the Internet provides rich multimedia interaction opportunities making consumers more willing to participate in product design [46]. Wang and Hao (2018) argued that Internet penetration facilitates the transition from environmental attitudes to participation in sustainable consumption [17]. Huang et al. (2022) used social media to explore public views and drivers of green consumption [47]. The above literature has mainly used micro data to explore the impact of Internet development or digital finance on household consumption. This paper differs from the above literature in that we use macro data for 30 Chinese provinces and focus on the impact of the penetration of Internet technologies into socio-economic activities on consumption upgrading.

### 2.3. The Impact of Consumption on Carbon Emissions

Consumption is the terminal manifestation of carbon emissions, and green consumption and low-carbon development have become a global consensus. Many scholars focus on the impact of household consumption on carbon emissions [48,49,50,51,52,53,54]. Some scholars have argued that the rapid growth of household consumption promotes increased carbon emissions [49,51,55]. For example, the overall growth of household consumption in Denmark is the key to promoting the increase in carbon emissions [56]. The structure of household consumption in China is one of the main factors contributing to household carbon emissions [48]. Carbon emissions from household consumption show significant differences among different income groups [48], with higher carbon emissions from higher-income households [57,58]. In contrast to the above findings, the main reason for the decrease in carbon emissions in the United States was the change in consumption patterns [59]. Household carbon emissions in China would be reduced under a scenario of low carbon consumption patterns [60]. With the refinement of carbon emissions calculation to the residential sector, scholars have begun to explore the impact of household consumption structure on residential carbon emissions [48,61,62]. However, the existing studies mainly reflect the impact of consumption on household and residential carbon emissions. They do not reflect the carbon emissions of overall economic activities. In China, the impact of consumption upgrading on regional carbon emissions is becoming emerging research [63,64]. Most of the above literature explores the direct impact of consumption on carbon emissions. Scholars have yet to explore the relationship between consumption upgrading as an indirect impact mechanism on carbon emissions.

The existing literature mainly focuses on the impact of Internet development on carbon emissions or the impact of Internet development on consumption upgrading. Most of the literature explores the impact of Internet development on carbon emissions through traditional transmission mechanisms [24,27,29]. This paper explores a new perspective on consumption upgrading. We include Internet development, consumption upgrading, and carbon emissions in a unified research framework. Therefore, this paper uses panel data for 30 Chinese provinces (except Tibet, Hong Kong, Macao, and Taiwan) from 2000 to 2018 to explore the impact of Internet development on carbon emissions from the perspective of consumption upgrading. We investigate the impact of Internet development on carbon emissions through consumption upgrading and verify the mediating role of consumption upgrading.

### 2.4. Theoretical Analysis and Research Hypothesis

#### 2.4.1. Internet Development and Carbon Emissions

There are three aspects of the negative externalities of Internet development on the environment. First, at the product level, people use end products such as smartphones, tablets, laptops, and smart TVs [2,21], and according to Life cycle theory, Life cycle assessment (LCA) of end products such as manufacturing and production energy consumption, service life, and recycling and disposal can lead to significant carbon emissions [2,21,65,66]. Second, the Internet has become an indispensable part of people’s daily life. According to the Interaction theory, it is known that the Internet has the advantage of being simple and easy to operate, which makes people using the Internet significantly increase in frequency. For example, people often use the Internet to communicate, maintain friendships, shop on e-commerce platforms, telecommute, purchase airline tickets and perform other daily behaviors, consuming a large amount of electricity, leading to an increase in carbon emissions. Third, with the rapid development of digital industrialization, data centers operating artificial intelligence, blockchain, cloud computing, big data, and other digital technologies, cause electricity consumption and infrastructure energy consumption to rise, which significantly contributes to the increase in carbon emissions. The increased demand and supply of ICT-intensive products will also lead to further energy consumption, thus contributing, to a certain extent, to the growth of carbon emissions [67]. Given the rapid growth of China’s Internet industry and its position as the world’s largest carbon emitter, we expect a positive correlation between Internet development and carbon emissions. Therefore, this hypothesis is proposed.

**Hypothesis** **1.** *Internet development significantly contributes to the impact of carbon emissions.*

#### 2.4.2. Internet Development and Consumption Upgrading

In the Internet era, material needs are constantly being satisfied, personalized and customized consumer needs are emerging, and this development of consumer values is in line with Maslow’s hierarchy of needs theory. Maslow’s hierarchy of needs theory [68] proposed that human needs can be divided into five categories: physiological needs, security needs, social needs, respect needs, and the need for self-actualization. Chinese consumer needs tend to expand from low-level material needs to high-level spiritual needs, resulting in consumption upgrading. 

The path of the Internet to promote consumption upgrading is mainly reflected in the following aspects. First, businesses improve the efficiency of resource allocation scientifically and rationally with the help of big data, cloud computing, and other information technologies, improve the efficiency of the industrial chain production, storage, circulation, and other links, promote the transformation of industrial structure, and develop diverse consumption areas thus promoting consumption upgrading. Second, enterprises understand consumer preferences through Internet consumer data, capture consumer feedback on products and services, and understand consumers’ personalized needs for products, thus promoting consumption upgrading. Third, promoting consumption upgrading from the consumption side. According to the theory of Consumer Behavior [69], the Internet maximizes the utility of consumer behavior by changing consumers’ behavior, decisions, and perceptions, which is conducive to increasing consumers’ willingness to purchase online and thus achieving consumption upgrading. Specifically, the Internet has significantly reduced consumers’ search and shopping costs and increased the transparency of information and prices across the market, making transactions more efficient and thus making consumers more inclined to use the Internet for consumption. At the same time, combined with Maslow’s Hierarchy of Needs theory, when human needs are satisfied at lower levels, they tend to pursue goods and services that bring happiness and enjoyment, focusing on spiritual satisfaction and comfort. This is reflected in the fact that the Internet gives consumers more choices of personalized goods and services, which makes the DEC account for a higher proportion of total consumption, thus promoting consumption upgrading. 

In addition, the development of the Internet has helped the market to bring about an apparent long-tail effect. Online shopping has broken companies’ time and space constraints to sell goods. With the development of e-commerce in China, the consumption scenario is not only simple online shopping, but most importantly, it brings cross-border integration of different virtual scenarios. Such as the combination of short video platforms and e-commerce brings a new shopping experience, which helps companies better respond to the long-tail phenomenon of demand, enabling more consumers to participate in online personalized consumption and promoting consumption upgrading. Therefore, this hypothesis is proposed.

**Hypothesis** **2.** *Internet development will promote consumption upgrading.*

#### 2.4.3. Consumption Upgrading and Carbon Emissions

Internet development has a significant impact on consumer upgrading through Internet technology penetration. After consumers purchase development and enjoyment goods, consumers’ personalized needs are fed back into the design, production, and packaging of goods, which in turn affects carbon emissions.

First, businesses are aware of consumers’ psychological activities. After consumers purchase more developmental and enjoyment-oriented goods, they give rise to enterprises to develop and produce more innovative products, forcing the production side to pay attention to personalized design and technological innovation capabilities, which in turn promotes industrial structure upgrading [70], thus affecting carbon emissions [71].

Second, businesses understand the attention economy and consumers’ psychological needs and reflect the advanced novelty of goods from their packaging design, and many consumers will consume the products because of their advanced packaging. In the long run, this has resulted in excessive packaging and high consumables of packaging materials, causing waste of resources [72], environmental pollution, and increased carbon emissions.

Third, as far as the consumption side is concerned, consumption upgrading has increased consumer spending on higher consumption levels, such as household equipment and supplies, transportation and communication, and health care. Consumption upgrading increases people’s fuel demand for transportation, heating, and electricity, which increases carbon emissions from energy consumption and thus affects CO_2_ emissions. Based on the above analysis, we tested the following hypotheses.

**Hypothesis** **3.** *Consumption upgrading has a catalytic effect on carbon emissions.*

**Hypothesis** **4.** *Internet development will indirectly impact carbon emissions through consumption upgrading.*

## 3. Methodology and Data

### 3.1. Model Setting

According to our previous discussion of transmission mechanisms, Internet development may affect carbon emissions through consumption upgrading. Mediating effect means that the explanatory variable influences the explained variable through one or several intermediate variables, which are called mediating variables [67,73,74]. Standard mediating effects model, shown as Equations (1)–(3).

(1) $$Y=\alpha X+\varepsilon_{1}$$

(2) $$M=\beta X+\varepsilon_{2}\;$$

(3) $$Y=\alpha^{\prime }X+\gamma \beta +\varepsilon_{3}$$

where *X* is the explanatory variable; *Y* is the explained variable; and *M* is the mediating variable.

This paper uses panel data, which have individual and temporal dimensions compared to cross-sectional data. Panel data can solve the problem of omitted variables while providing information on the dynamic behavior of individuals. First, according to Equation (1) extended to Equation (4), based on the regression estimation of the impact of Internet development ($Internet_{it}$) on carbon emissions ($perCO_{2it}$), this paper establishes the baseline regression to test Hypothesis 1.

(4) $$perCO_{2it}=\alpha_{0}+\alpha_{1}Internet_{it}+\alpha_{2}control_{it}+\varepsilon_{it}$$

Second, according to Equation (2) extended to Equation (5), we examine the impact of Internet development ($Internet_{it}$) on consumption upgrading ($cu_{it}$) to test Hypothesis 2.

(5) $$cu_{it}=\beta_{0}+\beta_{1}Internet_{it}+\beta_{2}control_{it}+\varepsilon_{it}$$

Finally, according to Equation (3) extended to Equation (6), carbon emissions ($perCO_{2it}$) as the explained variable, Internet development ($Internet_{it}$) as the explanatory variable, and consumption upgrading ($cu_{it}$) as the mediating variable were put into the model for regression estimation to test Hypotheses 3 and 4.

(6) $$perCO_{2it}=\gamma_{0}+\gamma_{1}Internet_{it}+\gamma_{2}cu_{it}+\gamma_{3}control_{it}+\varepsilon_{it}$$

where variable $perCO_{2it}$ represents per capita CO_2_ of the province *i* in year *t*; variable $Internet_{it}$ represents the internet development level of the province *i* in year *t*; variable $\;cu_{it}$ represents consumption upgrading of the province *i* in year *t*; $control_{it}$ is a series of control variables; and $\varepsilon_{it}\;$ are random disturbance terms.

According to the principle of the mediating model test, the coefficient $\alpha_{1}$ measures the total effect of Internet development on per capita carbon emissions and $\alpha_{1}$ is expected to be significantly positive. The coefficient $\beta_{1}$ measures the effect of Internet development on consumption upgrading and $\beta_{1}$ is expected to be significantly positive. The coefficient $\gamma_{1}$ measures the direct effect of Internet development on per capita carbon emissions, and the product $\gamma_{2}\beta_{1}$ of the coefficient $\gamma_{2}$ and the coefficient $\beta_{1}$ measures the mediating effect of consumption upgrading, i.e., Internet development affects per capita carbon emissions by promoting consumption upgrading. The absolute value of the expectation coefficient $\gamma_{1}$ is smaller than the absolute value of the coefficient $\alpha_{1}$, i.e., consumption upgrading partially mediates the promotion of carbon emissions by Internet development.

### 3.2. Variable Selection

Considering the availability and scientific nature of the data, this paper selected data from 30 provinces in China (excluding Tibet, Hong Kong, Macao, and Taiwan) for the period from 2000 to 2018, as the data on Internet penetration in Chinese provinces are only available up to 2018, and 2019–2020 are not published. Other data are mainly from the statistical yearbooks of 30 provinces from 2000 to 2018. 

#### 3.2.1. Explained Variable

**Figure 2:**
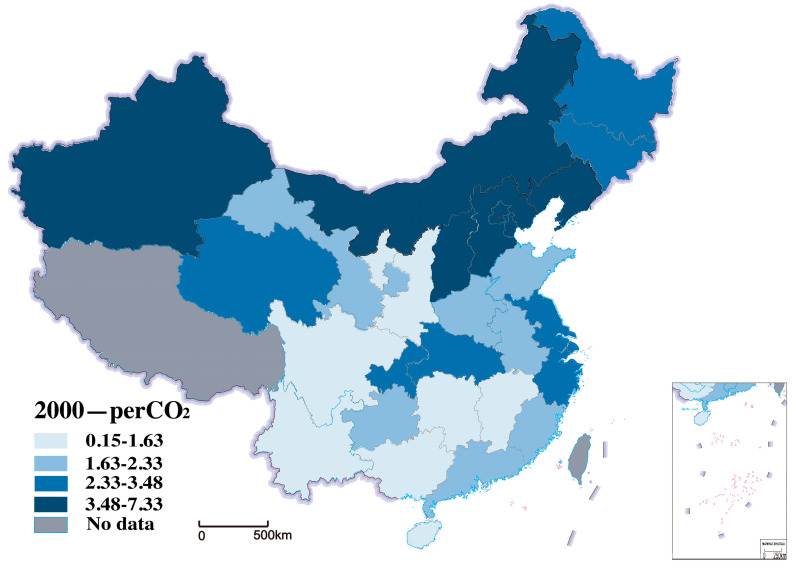
Distribution of carbon emissions per capita in 2000.

**Figure 3:**
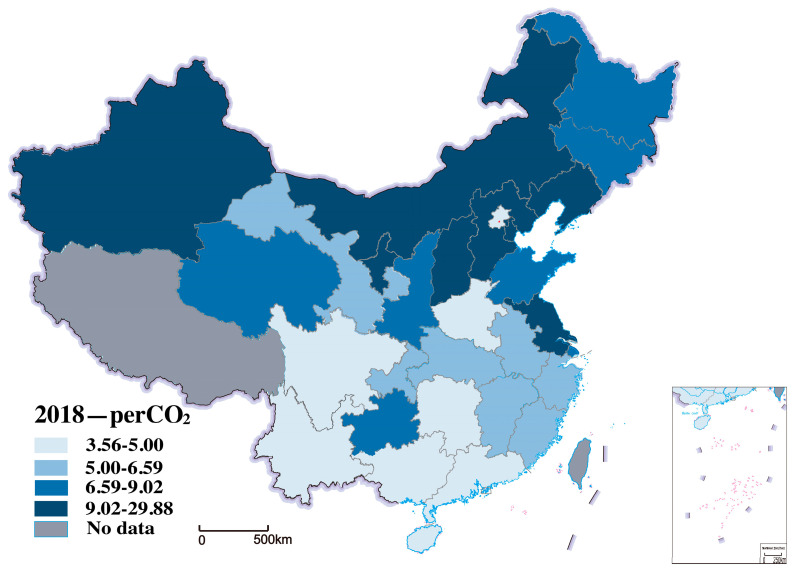
Distribution of carbon emissions per capita in 2018.

Carbon emissions are the explained variable. We use per capita CO_2_ to characterize. Since China’s carbon emissions are mainly CO_2_, this study uses per capita CO_2_ to measure carbon emissions. According to China’s provincial carbon dioxide emissions inventory, CO_2_ emission was calculated by the Intergovernmental Panel on Climate Change (IPCC) sectoral methodology and CO_2_ emissions data from the database of China Emissions Accounts and Datasets (CEADs) [75]. Figure 2 shows the distribution of carbon emissions per capita in 2000 in the study area, and Figure 3 shows the distribution of carbon emissions per capita in 2018. We can see that the carbon emissions per capita have increased from 2000 to 2018 in all 30 provinces, as shown in Figure 2 and Figure 3.

#### 3.2.2. Explanatory Variable

**Figure 4:**
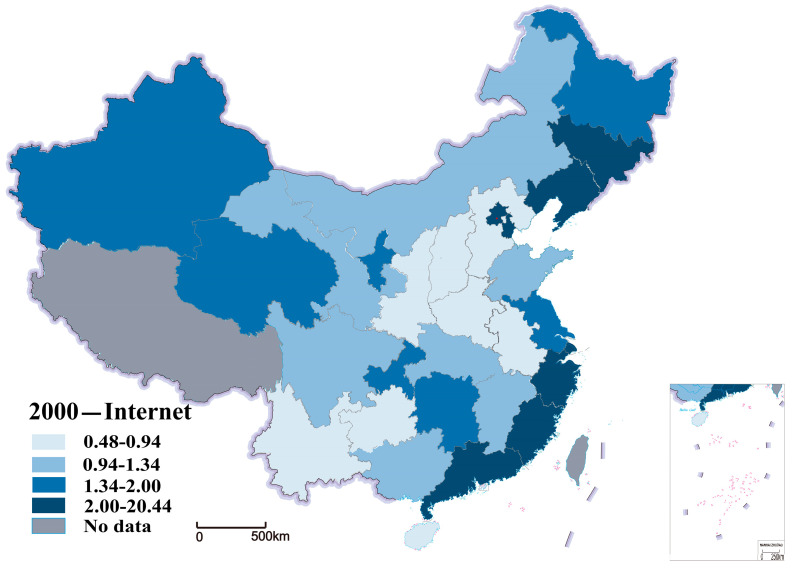
Distribution of Internet development in 2000.

**Figure 5:**
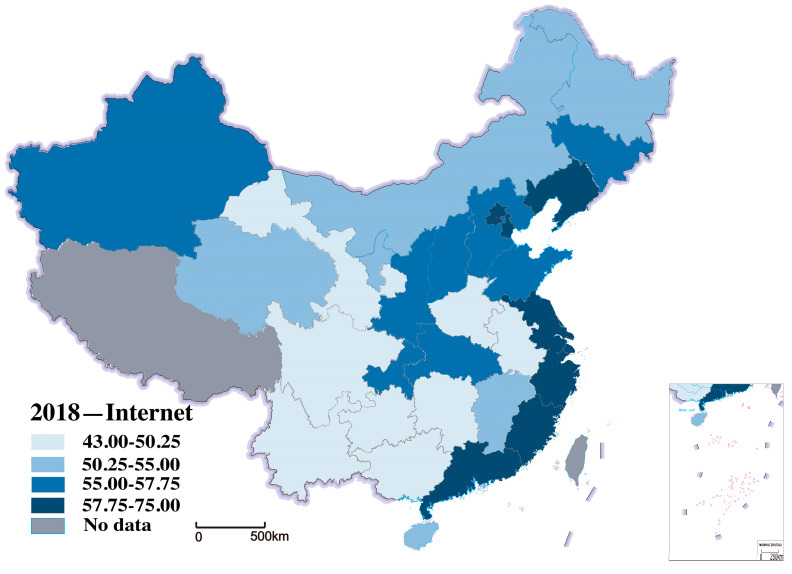
Distribution of Internet development in 2018.

Internet development level is the explanatory variable, referenced from the China Internet Network Information Center (CNNIC), which is commonly used in the existing literature. We use Internet penetration to measure the level of internet development in a region, which has the advantage of simple calculation and precise meaning. Figure 4 shows the distribution of Internet development in 2000 within the study area. Figure 5 examines the distribution of Internet development within the region in 2018. We can see a significant increase in Internet development in 30 provinces from 2000 to 2018, as shown in Figure 4 and Figure 5. 

#### 3.2.3. Mediating Variable

**Figure 6:**
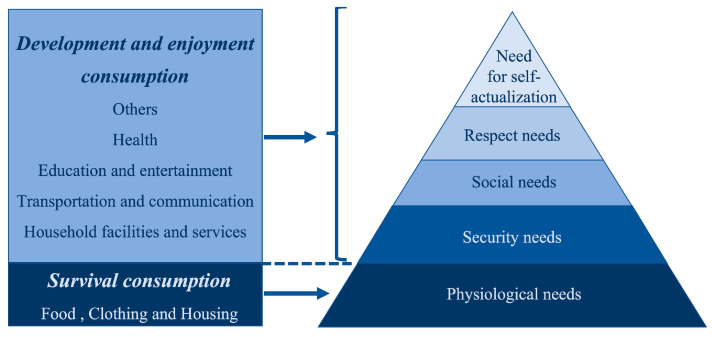
Consumption category and Maslow’s hierarchy of needs.

Consumption upgrading is the mediating variable. *The China Statistical Yearbook* classifies consumption expenditure into eight main categories: food, clothing, housing, household facilities and services, transportation and communication, education and entertainment, health, and others, as shown in Figure 6. The first three types of consumption expenditure are classified as SC [76], which can only provide people with the production and living materials needed for survival and can only satisfy low-level physiological needs in Maslow’s Hierarchy of Needs. The last five types of consumption are classified as DEC, which can satisfy people’s spiritual needs and enhance their happiness after satisfying their physiological needs. In this paper, we manually collect data on eight consumption expenditures from 2000 to 2018 in 30 provinces and use the ratio of DEC in total consumption to measure consumption upgrading. When consumption upgrading is in the interval (0, 1) and the value is closer to 0, the lower the DEC ratio, indicating a lower degree of consumption upgrading. When the value is closer to 1, the higher the DEC ratio, and the higher the degree of consumption upgrading.

#### 3.2.4. Control Variable

(1) Population size (size). There is a significant difference in population size among Chinese provinces and the difference in population size inevitably affects regional carbon emissions. Some studies have shown that population size is one of the drivers of carbon emissions [77,78]. An increase in population size leads to an increase in production and consumption demand, and people’s living and consumption activities have an impact on carbon emissions through population clustering [12]. In this paper, the year-end resident population in each province is chosen to characterize the population size.
(2) Economic development level (perGDP). China has a vast territory and the level of economic development varies significantly from province to province. Since there is a significant correlation between economic development and carbon emissions [67,79], we need to control the impact of economic development on carbon emissions. In this paper, we choose the GDP per capita of each province to characterize the regional economic development level.
(3) Industrial structure (ind). China’s secondary industrial development is dominated by fossil energy consumption (e.g., coal and oil) and industrial development brings a large amount of energy consumption as one of the crucial sources of carbon emissions [27]. The industrial structure varies across Chinese provinces and some studies have shown that the impact of industrial structure on carbon emissions shows a significant correlation [67,80], which is measured by choosing the secondary sector gross product ratio to GDP in this paper. See the descriptive statistics of the variables in Table 1 for details.

## 4. Empirical Results

### 4.1. Regression Results

This paper uses Hausman test panel data and gets a test result of a *p*-value less than 0.05, which indicates that this paper should use the fixed effects model for analysis. At the same time, because the provinces are different in economic development, Internet development, and consumer market, and individual differences are apparent, the analysis from the theoretical level, we also use the fixed effects model to make the judgment. 

We estimated Equations (4)–(6) using stata16 software, and the results are presented in Table 2. To test Hypothesis 1, in column (1), we used the Internet penetration rate to represent the regional Internet development level to estimate its effect on carbon emissions. The results show that Internet development and carbon emissions are significantly positive at the 5% level ($\alpha_{1}\;$= 3.885, with a total effect coefficient of 3.885). When all other parameters remained equal, the average per capita carbon emissions increased by 3.885 t for every 1.000% increase in the Internet development level. Thus, the development of the Internet could promote per capita CO_2_, and Hypothesis 1 is verified. 

To test Hypothesis 2, the results in column (2) indicate that Internet development is significantly related to consumption upgrading ($\beta_{1}\;$= 0.026), which is significant at the 1% level. When all other parameters remained equal, the average consumption upgrading increased by 0.026% for every 1.000% increase in Internet development. Increasing the Internet development level contributed to consumption upgrading. This finding is consistent with the conclusions and Hypothesis 2 is verified. 

To test Hypothesis 3, column (3) results indicate the mediating effect of consumption upgrading between Internet development and carbon emissions. The level of Internet development ($\gamma_{1\;}$= 3.433, direct effect coefficient 3.433.) is significant at the 5% level. Every 1% increase in Internet development is accompanied by a 3.433 t increase in per capita CO_2_. The coefficient of consumption upgrading ($\gamma_{2}$ = 17.125) is significant at the 5% level, indicating that each 1% increase in consumption upgrading leads to a 17.125 t increase in carbon emissions, which means that consumption upgrading significantly contributes to the increase in carbon emissions. Hypothesis 3 is verified.

To test Hypothesis 4, comparing the coefficients of Internet development in columns (1) and (3), the absolute value of $\gamma_{1}$ in column (3) is smaller than the absolute value of $\alpha_{1}$ in column (1). According to Equation (6), the regression coefficient for the impact of Internet development on carbon emissions decreased from 3.885 t to 3.433 t after adding consumption upgrading as a mediating variable, thereby indicating that the Internet development increased the per capita carbon emissions through its positive impact on consumption upgrading. The proportion of the indirect effect ($\beta_{1}\gamma_{2}$) to the total effect ($\alpha_{1}$) is 11.46%. Hypothesis 4 is verified. 

In this paper, the Sobel test is used to validate the mediating effect model further to ensure the reliability of the findings. As shown in Table 2, three significance tests are provided in the Sgmediation command test process, namely the Sobel test, Goodman1 test, and Goodman2 test, all of which are significant at the 1% level, indicating that the findings support the above four hypotheses. The Sobel test method’s direct effect coefficients, total effect coefficients, and indirect effect ratios are 3.433, 3.885, and 11.6%. This conclusion is consistent with the results of the Three-Step Method, which further illustrates the reliability of the mediation effect model.

### 4.2. Robustness Tests

The previous section focuses on the transmission mechanism of Internet development on carbon emissions, and this section further conducts Bootstrap tests on the previous results. The endogeneity problem may arise in identifying the impact of the Internet on carbon emissions. We regress the lagged term of the Internet to mitigate the reverse causality problem while selecting instrumental variables and using two-stage least squares (2SLS) to mitigate the endogeneity problem.

#### 4.2.1. Bootstrap Method Test

This paper uses the Bootstrap method to verify the transmission mechanism of consumption upgrading more rigorously between Internet development and carbon emission. As shown in Table 3, the results in Table 3 indicate that the indirect effect and direct effect are significant at the 1% level, respectively, with the indirect effect accounting for 11.63%. The 95% confidence interval of the indirect effect is [0.230, 0.787], which does not contain 0, which indicates that consumption upgrading is the mediation effect of Internet development and carbon emissions. The 95% confidence interval of the direct effect is [2.442, 4.615], which does not contain 0. This conclusion indicates that Internet development has a direct effect on carbon emissions. The Indirect effect coefficient is 0.452 and the direct effect coefficient is 3.433. The above conclusion is consistent with the results of the Three-Step method and the Sobel test method. It further verifies the transmission mechanism of consumption upgrading between Internet development and carbon emissions.

#### 4.2.2. Replaced the Explanatory Variable

To ensure the reliability of the empirical results, we replaced the explanatory variable (Internet) with software business revenue (sw), the source of this indicator is the *China Electronic Information Industry Statistical Yearbook*. Software is the basis of Internet economic development, the link between information technology and consumption terminal integration, and the carrier of Internet consumption. Higher software business revenue (sw) indicates deeper integration of Internet development and industry. As shown in Table 4, the first column shows the regression results with the effect of Internet development on carbon emissions, which indicates that the coefficient of the explanatory variable (sw) is 1.151, which is significantly positive at the 5% level, which fully indicates that Internet development significantly contributes to carbon emissions; the second column shows the effect of Internet development (sw) on consumption upgrading (cu), the coefficient of (sw) is 0.004, which is positive at 10% significance level, which indicates that Internet development significantly promotes consumption upgrading. The third column demonstrates the effect of Internet development and consumption upgrading on carbon emissions. The results show that the (sw) coefficient is 1.085, which is significantly positive at the 5% level, and the consumption upgrading coefficient is 18.389, which is significantly positive at the 5% level, which verifies that Internet development affects carbon emissions through consumption upgrading. Table 4 is generally consistent with the results of the analysis in Table 2, which proves the robustness of the conclusion.

#### 4.2.3. Endogenous Test

The problem of endogeneity is a necessary issue in economic research. From the focus of this paper, there are two possible endogeneity issues. First, there may be a mutual causality between Internet development and carbon emissions. Second, many factors affect carbon emissions and the explanatory variable involved in the current data may create the problem of omitted variables. 

In this paper, a lagged period of the Internet development level is selected for regression to address the mutual causality between Internet development and carbon emissions. We can see in Table 5 that the significance of the model results in columns (1) to (3) being consistent with the significance of the results in Table 2.

In this paper, we selected instrumental variables and used the two-stage least squares method to solve the endogeneity of omitted variables problem. Based on the reference, the spherical distance (distance) from 30 provincial capital cities to Hangzhou is selected as an instrumental variable for Internet development in conjunction with this paper to satisfy the exogeneity and correlation requirements of the instrumental variable. The spherical distance from the provincial capital cities to Hangzhou is not correlated with carbon emission; while Hangzhou is one of the cities with the best Internet economy development in China, and the radiation drive of the Internet economy is correlated with distance and then there is a correlation between Internet development and the instrumental variable. In addition, considering that the spherical distance (distance) does not vary with time, drawing on [12,81,82], we interact the spherical distance (distance) with the one-period lag term of Internet development (L1.Internet) to construct a new instrumental variable (distance*L1.Internet) that varies with time.

The 2SLS regression results are shown in Table 6. Column (1) presents instrumental variables significant at a 1% statistical level, satisfying the instrumental variables correlation requirement, and the coefficient of the instrumental variable is positive, indicating that the instrumental variables show a positive correlation with Internet development, which is consistent with the expected exploration results. The first stage F statistic (Cragg-Donald Wald F) is significantly greater than 10, indicating no weak instrumental variable problem. In terms of coefficients, the second stage regression results indicate that the Internet development coefficient is significantly positive at the 1% level, which further supports the conclusion of Hypothesis 1, indicating that the conclusion that Internet development significantly contributes to per capita carbon emissions still holds after mitigating the potential endogeneity. In addition, this paper also tests the under-identification of instrumental variables (Anderson Canon LM), and the *p*-value is less than 0.01, which is significant at the 1% level, indicating that the original hypothesis of “under-identification of instrumental variables” is rejected. Therefore, the instrumental variables selected in this paper are reasonable and valid.

### 4.3. Heterogeneity Analysis

China is a vast country with many provinces, with regional differences in Internet development and carbon emissions. We divided the 30 provinces of mainland China (except Tibet) into three parts: eastern, central, and western according to the regional division criteria of China (see Appendix A for the provinces in each region) and ran regressions according to Equations (4)–(6) to analyze their regional heterogeneity. As shown in Table 7, we can see from columns (1) to (3) that Internet development in the eastern region significantly promotes carbon emissions and the coefficient of the Internet is 1.24, significant at the 5% level. Internet development promotes consumption upgrading and the coefficient of Internet development is 0.02, significant at the 5% level. Internet development and consumption upgrading promote the increase in carbon emissions and the coefficient of Internet development is 1.04, significant at the 10% level, and the coefficient of consumption upgrade is 9.15, significant at the 1% level. After adding the consumption upgrading mediating variable, the coefficient of Internet development decreases from 1.24 to 1.04, which proves that Internet development promotes the increase in carbon emissions through consumption upgrading. The results in columns (4) to (6) of Table 7 show that Internet development and consumption upgrading are insignificant in the central region. The results in columns (7) to (9) of Table 7 indicate that Internet development in the western region has no significant effect on carbon emissions and Internet development inhibits consumption upgrading.

The above results analyze that Internet development promotes carbon emissions through consumption upgrading, and then regions with high and low levels of Internet development may lead to significant differences in the results. There are many provinces in China with considerable differences in Internet development. The data distribution clearly shows the high and low levels of Internet development in 30 Chinese provinces (except Tibet and Hong Kong, Macao, and Taiwan), therefore we divide them into two types of regions according to the level of Internet development, the leading and lagging regions of Internet development (see Appendix B for the provinces in the two types of regions). The heterogeneity of the leading and lagging regions of Internet development is analyzed by regression according to Equations (4)–(6). As shown in Table 8, columns (1)–(3) show that Internet development in the leading region significantly promotes carbon emissions, and the Internet coefficient is 1.901, which is significant at the 1% level; Internet development promotes consumption upgrading and the consumption upgrading coefficient is 0.015, which is significant at the 5% level. Internet development and consumption upgrading promote increased carbon emissions and the coefficient of Internet development is 1.673, significant at the 1% level, and the coefficient of consumption upgrading is 14.841, significant at the 10% level. After adding the consumption upgrade mediating variable, the coefficient of Internet development decreases from 1.901 to 1.673, proving that the Internet development level contributes to the increase in carbon emissions through consumption upgrade. The results in columns (4)–(6) of Table 8 show that the effects of Internet development and consumption upgrading on carbon emissions in the lagging region are insignificant.

## 5. Discussion

Global climate change results from more than a century of unsustainable lifestyles, consumption, and production patterns. Therefore the right policies, infrastructure, and technologies need to be put in place to enable a change in the way we live and behave. By 2050, greenhouse gas emissions will be reduced by 40–70% (IPCC, 2022). Therefore, carbon reduction in Internet infrastructure and data centers should be encouraged and low carbon consumption in people’s daily life should be emphasized. This paper focuses on a new perspective of consumption upgrading, which differs from most scholars who focus on traditional transmission mechanisms [7,12,13,24,27,29,83]. We put Internet development, consumption upgrading, and carbon emission in a unified research framework. We focus on the theoretical mechanisms underlying Internet development, consumption upgrading, and carbon emissions and empirically test consumption upgrading as a mediating effect between Internet development and carbon emissions.

We find that Internet development promotes an increase in carbon emissions. This finding is consistent with [27,28,83]. There are two possible reasons to explain this finding. First, the explosive growth of the Internet economy has led to the investment of more resources in Internet infrastructure and increasing energy consumption in data centers [84], which affects carbon emissions. Second, the penetration of the Internet into the entire socio-economy and the inseparability of society’s development, people’s production, and life from the Internet lead to a large amount of electricity and energy consumption, which inevitably contributes to the growth of carbon emissions.

We find that Internet development has a catalytic effect on consumption upgrading. This conclusion is consistent with the findings of studies [45,85]. We also verified this finding based on Maslow’s hierarchy of needs theory. There are three possible reasons for revealing this conclusion: First, the development of the Internet has increased the transparency of information about goods. Online shopping has the advantages of easier comparison of product prices and features [45] and saving time in selecting goods, and consumers are more willing to shop online to meet their individual needs, thus promoting consumption upgrading. Second, the Internet development has changed how Chinese consumers pay. Chinese consumers do not need to use cash to purchase goods online or offline; digital payment improves the convenience of payment, enhances consumption efficiency, and stimulates consumption demand to enhance consumption upgrading. Thirdly, the Internet consumption model is supplementing and partially replacing offline physical consumption, and with the vital radiation function of Internet consumption, consumers’ spending on DEC has increased significantly, effectively achieving consumption upgrading.

We find that consumption upgrading significantly affects carbon emissions, a result that shares with [61]. The possible reasons for revealing this finding are as follows. First, consumption upgrading causes people to consume more goods and services such as household equipment and supplies, transportation and communication, education, culture and entertainment, and health care daily, which consumes more energy and thus affects the increase in carbon emissions. Second, consumption upgrading will lead to more enterprises producing development and enjoyment goods that meet consumers’ diversified and personalized needs, which will affect the increase in carbon emissions. Consumption upgrading is an indirect effect of Internet development on carbon emissions. Online shopping can break the geographical distance limitation, and the rapid development of e-commerce has given rise to the express delivery industry, where over-packaged goods may be one of the factors leading to the increase in carbon emissions [86]. In addition, the maturing technology of Internet development and the penetration of mobile devices has led to an increase in the number of personalized goods purchased by consumers, resulting in the prevalence of online shopping [75], and the complex online environment has led to the creation of impulsive purchasing desire by consumers. Overconsumption may be one of the important factors contributing to the increase in carbon emissions [87].

There are apparent differences in the impact of Internet development and consumption upgrading on carbon emissions among these regions. On the one hand, as far as the level of Internet development is concerned, the rapid growth of the Internet economy in the eastern and leading regions, especially the radiation-driven effect of the Internet economy in cities such as Hangzhou and Shenzhen, has led to higher Internet development in the two regions. People in the eastern and leading regions will increase the frequency of using the Internet for social behaviors such as remote communication, online shopping, and telecommuting. However, the carbon-intensive sectors that bear the operation of the Internet (e.g., electricity) consume more energy and may cause significant carbon emissions. The level of economic development in the central region is lower than eastern region. The central region lacks large Internet enterprises, which leads to a lower level of Internet development in the central region than in the eastern region. The western region is vast and has a small population, but the Internet infrastructure still needs to be completed. It is not closely connected to the eastern and central regions due to factors such as topography and climate, resulting in relatively backward Internet development. On the other hand, as far as consumption is concerned, due to the underdeveloped economy in the western region, people spend more on SC, such as daily clothing, food, and housing, which leads to a lower degree of consumption upgrading. The eastern and leading regions have a higher level of economic development and people’s SC is satisfied. Consumers tend to buy more DEC products (such as household equipment and supplies, transportation and communication, education, culture and entertainment, health care, and other types of consumption), and consumption is the terminal of carbon emission, so the consumption upgrading in the eastern and leading regions promote the increase in carbon emissions.

The findings of this paper provoke us to reflect that most policymakers and scholars strongly encourage the integration and development of various industries with Internet technologies, which may only partially be conducive to environmental sustainability and better human life. The future Internet industry needs to follow the same green and low-carbon principles for high-quality development as other energy-consuming industries, and the Internet industry needs to reduce emissions from the energy consumption side of data centers. Meanwhile, the ultimate purpose of production is consumption, and people’s consumption behavior is the end point of carbon emission. The full use of idle resources can reduce carbon emissions. Consumers or enterprises can use the Internet to integrate idle resources and give up the right to use idle resources to others for a fee. By sharing idle resources with others, the resources are fully utilized, thus reducing energy consumption and carbon emissions. Green and low-carbon consumption behavior is a crucial measure to reduce end carbon emissions, and consumers also need to focus on a simple and moderate lifestyle and consumption.

The study currently suffers from the following two shortcomings. First, the conclusion of our study that Internet development promotes the growth of carbon emissions may be the result of a short-term impact and the results may be different if the long-term impact between the two is explored. We will consider verifying the long-term impact relationship of Internet development on carbon emissions in future studies. Second, we did not explore the spatial spillover effect due to the obvious spatial differences among the Chinese provinces. We will explore regional spatial heterogeneity and spatial spillover effects in future studies. 

## 6. Conclusions and Policy Recommendations

### 6.1. Conclusions

This paper uses Maslow’s hierarchy of needs theory and consumer behavior theory to analyze consumption upgrading, Internet development, and carbon emissions in a unified research framework. Based on the panel data of 30 provinces in China, we try to clarify the relationship between consumption upgrading, Internet development, and carbon emissions and explore the mediating effect of consumption upgrading between Internet development and carbon emissions using the fixed effects model and mediating effects model and reveal the effect of Internet development on carbon emissions. This paper provides references for achieving China’s “double carbon” goal. The main conclusions of this paper are as follows:

(1) Internet development will contribute to the increase in carbon emissions. The higher the level of Internet development, the higher the DEC ratio (consumption upgrading), i.e., Internet development promotes consumption upgrading;
(2) Consumption upgrading is a mediating effect between Internet development and carbon emissions. When the consumption upgrade mediating variable is added, the coefficient of Internet development level decreases, further indicating that consumption upgrade plays a partial mediating role in Internet development and carbon emission;
(3) Internet development and consumption upgrading in the eastern region contribute significantly to carbon emissions, while they are not significant in the central and western regions. The transmission mechanism between Internet development and carbon emissions is consumption upgrading. The Internet development leading region promotes carbon emissions through consumption upgrading. In comparison, the lagging region has no significant effect on consumption upgrading and carbon emissions.

### 6.2. Policy Recommendations

Based on the above conclusions, the following policy recommendations are proposed. 

First, the government should focus on cultivating people’s correct consumption concept. The carbon emission generated by the consumption terminal has become a problem that the government, enterprises, and everyone cannot ignore, so it is urgent to promote moderate and simple consumption. The government should reasonably use new media, short videos, and other information platforms to spread the concept of healthy and green consumption to consumer groups and cultivate a rational and moderate consumption concept among consumers.

Second, the government should develop a reward and punishment system to promote the green transformation of commodity packaging. Designers should consider the relationship between the design and the natural environment in the commodity packaging design, avoid excessive packaging and reduce waste, use environmentally sustainable and degradable environmental protection materials, save resource consumption, and promote the recycling of packaging again.

Third, China and other countries should pay great attention to the carbon emissions of Internet development itself when vigorously developing the Internet economy, guide the Internet industry to raise awareness of energy conservation and emission reduction, encourage the Internet industry to take the social responsibility of green development and promote other industries’ green and intelligent transformation, and actively explore the path of sustainable low-carbon development and promote the country to achieve the “double carbon” goal. 

## Figures and Tables

**Table 1:** Descriptive statistics of variables.

Variable	Mean	Std. Dev.	Min	Max
perCO_2_	6.213	4.353	0.126	29.875
Internet	2.973	1.070	0.392	4.369
cu	0.400	0.033	0.310	0.539
ind	0.455	0.081	0.186	0.664
perGDP	10.074	0.854	7.887	11.851
size	8.160	0.758	6.249	9.421

Note: variables, such as cu and ind are expressed in ratios and other variables are nature logarithms.

**Table 2:** Regression results.

	(1)	(2)	(3)
Variable	perCO_2_	cu	perCO_2_
Internet	3.885 **	0.026 ***	3.433 **
	(2.657)	(2.840)	(2.710)
cu			17.125 **
			(2.226)
Control variables	YES	YES	YES
Constant	−104.011	1.484 ***	−129.426
	(−1.393)	(3.213)	(−1.650)
R-squared	0.611	0.401	0.624
Time effects	YES	YES	YES
Province effects	YES	YES	YES
Observations	570	570	570
Number	30	30	30
Sobel test	0.452 *** (z = 2.957)		
Goodman test1	0.452 *** (z = 2.915)		
Goodman test2	0.452 *** (z = 3.000)		
Indirect effect coefficient	0.452		
Direct effect coefficient	3.433		
Total effect coefficient	3.885		
Indirect effect ratio	0.116		

Note: *** and ** indicate *p* < 0.01 and *p* < 0.05; t-statistics in parentheses.

**Table 3:** Bootstrap test of consumption upgrading.

Effect	Estimated Coefficient	S. E.	Z-Value	95% Confidence Interval	
Indirect Effect	0.452 ***	0.153	2.961	0.230	0.787
Direct Effect	3.433 ***	0.709	4.842	2.442	4.615

Note: *** indicates *p* < 0.01.

**Table 4:** Robustness results.

	(1)	(2)	(3)
Variable	perCO_2_	cu	perCO_2_
sw	1.151 **	0.004 *	1.085 **
	(2.454)	(2.011)	(2.337)
cu			18.389 **
			(2.073)
Control variables	YES	YES	YES
Constant	−80.245	1.736 ***	−112.177
	(−1.149)	(4.516)	(−1.408)
R-squared	0.623	0.387	0.639
Time effects	YES	YES	YES
Province effects	YES	YES	YES
Observations	570	570	570
Number	30	30	30

Note: ***, ** and * indicate *p* < 0.01, *p* < 0.05 and *p* < 0.1; t-statistics in parentheses.

**Table 5:** Endogenous test results.

	(1)	(2)	(3)
Variables	perCO_2_	cu	perCO_2_
L1.Internet	3.436 ***	0.036 ***	2.827 ***
	(3.017)	(3.812)	(2.882)
cu			17.036 **
			(2.082)
Control variables	YES	YES	YES
Constant	−79.092	1.526 ***	−105.085
	(−1.208)	(3.165)	(−1.495)
R-squared	0.594	0.400	0.609
Province effects	YES	YES	YES
Year effects	YES	YES	YES
Observations	540	540	540
Number	30	30	30

Note: *** and ** indicate *p* < 0.01 and *p* < 0.05; t-statistics in parentheses; L1.Internet is a lag variable for one period.

**Table 6:** 2SLS regression.

	(1)	(2)
Variables	Internet	perCO_2_
Internet		8.630 ***
		(6.15)
Distance*L1.Internet	1.047 ***	
	(8.63)	
Control variables	YES	YES
Province effects	YES	YES
Year effects	YES	YES
Observations	570	570
Number	30	30
First Stage F-stat		74.408

Note: *** indicates *p* < 0.01; t-statistics in parentheses. L1.Internet is a lag variable for one period.

**Table 7:** Heterogeneity impact results.

	Eastern Region			Central Region			Western Region		
	(1)	(2)	(3)	(4)	(5)	(6)	(7)	(8)	(9)
Variables	perCO_2_	cu	perCO_2_	perCO_2_	cu	perCO_2_	perCO_2_	cu	perCO_2_
Internet	1.24 **	0.02 **	1.04 *	−1.03	−0.01	−1.11	0.94	−0.02 *	1.11
	(2.35)	(2.50)	(1.97)	(−0.51)	(−0.68)	(−0.55)	(0.70)	(−2.01)	(0.78)
cu			9.15 ***			−14.30			8.08
			(4.26)			(−1.15)			(0.63)
Control variables	YES	YES	YES	YES	YES	YES	YES	YES	YES
Constant	51.31	1.75 ***	35.26	−57.85	1.76 *	−32.74	−329.75 **	−0.73	−323.84 **
	(1.79)	(3.73)	(1.32)	(−0.50)	(1.94)	(−0.30)	(−2.67)	(−1.30)	(−2.72)
R-squared	0.84	0.24	0.85	0.60	0.61	0.60	0.74	0.48	0.74
Province effects	YES	YES	YES	YES	YES	YES	YES	YES	YES
Year effects	NO	NO	NO	NO	NO	NO	NO	NO	NO
Observations	209	209	209	152	152	152	209	209	209
Number	11	11	11	8	8	8	11	11	11

Note: ***, ** and * indicate *p* < 0.01, *p* < 0.05 and *p* < 0.1; t-statistics in parentheses.

**Table 8:** Regional heterogeneity analysis of Internet development level.

	Leading Region			Lagging Region		
	(1)	(2)	(3)	(4)	(5)	(6)
Variables	perCO_2_	cu	perCO_2_	perCO_2_	cu	perCO_2_
Internet	1.901 ***	0.015 **	1.673 ***	−0.606	−0.012	−0.545
	(4.361)	(2.167)	(3.969)	(−0.455)	(−1.230)	(−0.402)
cu			14.841 *			5.008
			(2.064)			(0.485)
Control variables	YES	YES	YES	YES	YES	YES
Constant	28.746	1.857 ***	1.191	−183.623	−0.128	−182.983
	(0.993)	(5.401)	(0.036)	(−1.114)	(−0.181)	(−1.127)
R-squared	0.748	0.241	0.771	0.555	0.440	0.556
Province effects	YES	YES	YES	YES	YES	YES
Year effects	NO	NO	NO	NO	NO	NO
Observations	304	304	304	266	266	266
Number	16	16	16	14	14	14

Note: ***, ** and * indicate *p* < 0.01, *p* < 0.05 and *p* < 0.1; t-statistics in parentheses.

## Data Availability

Not applicable.

## Appendix A

The eastern region includes Beijing, Tianjin, Hebei, Shanghai, Liaoning, Jiangsu, Zhejiang, Fujian, Shandong, Guangdong, and Hainan; the central region includes Shanxi, Jilin, Heilongjiang, Anhui, Jiangxi, Henan, Hubei, and Hunan; the western region includes Inner Mongolia, Guangxi, Chongqing, Sichuan, Guizhou, Yunnan, Shaanxi, Gansu, Qinghai, Ningxia, and Xinjiang.

## Appendix B

The leading area includes Beijing, Tianjin, Hebei, Shanghai, Liaoning, Jiangsu, Zhejiang, Fujian, Shanxi, Shandong, Guangdong, Shaanxi, Chongqing, Hubei, Jilin, and Xinjiang; the lagging area includes Heilongjiang, Ningxia, Inner Mongolia, Anhui, Jiangxi, Henan, Hunan, Guangxi, Sichuan, Guizhou, Yunnan, Gansu, Qinghai, and Hainan.
